# The Influence Factors of Psychological Understanding and Behavior Choice for Legal Industry Entrepreneurs Based on Artificial Intelligence Technology

**DOI:** 10.3389/fpsyg.2020.01615

**Published:** 2020-07-21

**Authors:** Zeyi Miao

**Affiliations:** School of Law, Southeast University, Nanjing, China

**Keywords:** entrepreneur, psychological understanding, behavior choice, law and artificial intelligence, behavior concentration

## Abstract

This study is aimed to promote China’s support for innovation and entrepreneurship in the context of the rapid development of the artificial intelligence industry. Considering the advance law-based governance implemented in this country, the relationship between the psychological understanding and behavior choice of entrepreneurs in the law industry and artificial intelligence industry was explored. The investigation was conducted in the form of questionnaire survey, and the data were collected by sending emails. In addition, the model was established to make the investigation direction clearer. The reliability analysis of the questionnaire was carried out by means of Cronbach α reliability coefficient. The validity analysis of the questionnaire was carried out by Kaiser-Meyer-Olkin (KMO) coefficient. The statistical software was used to analyze the correlation between the factors in the investigation. The results show that there is a positive correlation between behavior speed in the psychological understanding of entrepreneurs in the legal industry as well as the entrepreneurial experience and entrepreneur behavior. The concentration of entrepreneurs’ behavior is negatively correlated with the entrepreneur’s industrial experience. The behavior speed and concentration of entrepreneurs in the artificial intelligence industry are negatively correlated with the age of entrepreneurs, but their entrepreneurial behavior and psychological understanding do not show correlation. The behavior speed and concentration of entrepreneurs in the artificial intelligence industry are positively correlated with product innovation, technological innovation, and market innovation. Therefore, the behavior choice of entrepreneurs in the legal industry are highly correlated with psychological understanding. The entrepreneurs’ psychological understanding and behavior choice in the field of artificial intelligence do not show a direct correlation but indirect correlation through indirect factor innovation opportunities. The investigation on entrepreneurs’ psychological understanding and behavior choice can provide important theoretical support for entrepreneurs in China’s legal industry and artificial intelligence industry.

## Introduction

With the support and encouragement of China’s “mass entrepreneurship and innovation” policy, the number of innovative entrepreneurs has increased significantly. Entrepreneurship is one of the major factors to promote economic development, and innovation is also the source of China’s economic development, so both are indispensable in the development of China’s economy and society (Hooshangi and Loewenstein, 2018; Romano et al., 2018). Why can some people start a business successfully? What do successful entrepreneurs have in common? The answers can be different. However, there is a common point–the recognition of entrepreneurs’ psychological capital ability affects the success of entrepreneurship (Digan et al., 2019). In addition to entrepreneurs, innovators are now the object of strong state support. However, compared with the process and means of innovation, the country or work unit pays more attention to the results of innovation (Lerner et al., 2019). Since the 18th National Congress of the Communist Party of China put forward comprehensively promoting law-based governance, the entrepreneurial situation has been favorable in China’s legal field.

With the advent of the 5G era, the development of emerging industries such as the Internet of Things, artificial intelligence, and the Industrial Internet has been gradually accelerated. Entrepreneurship “fertile soil” in various industries has become more extensive. As one of the world’s top three cutting-edge technologies, artificial intelligence technology is naturally valued by the country. Therefore, the industries related to artificial intelligence have also expanded rapidly in recent years (Khurosani, 2018; Nikolaev et al., 2018; Ramadi et al., 2019; Zheng et al., 2019). Entrepreneurship activities have a huge effect on the economy and society, yet the world is facing turbulent economic and political environments now. The entrepreneurial enterprises still faces serious development problems, among which “high failure rate” has become the sword of Damocles hanging above the entrepreneurial world. Evidently, there is an urgent need to theoretically explore the key elements and influencing mechanisms that contribute to the improvement of entrepreneurial performance of enterprises (Wu et al., 2018). In the past, the exploration of the treasure behind failure was neglected due to “survivor deviation.” In the only relevant investigations, scholars also lacked the exploration on the precognition and decision-making mechanism that affected the formation of specific recovery content of enterprises. Therefore, it is difficult to explain the difference between behavior and performance between subjects.

Innovation and entrepreneurship have always been an important means to alleviate employment pressure, promote economic development, and stimulate technological innovation. As a result, how to stimulate the public’s entrepreneurial enthusiasm and encourage more people to enter the entrepreneurial team has become an urgent problem to be solved in all countries of the world. At the same time, more women are also actively involved in entrepreneurial activities; most of the current academic investigations on entrepreneurial behavior of entrepreneurs is based on worldwide results, lacking explorations on entrepreneurial behavior rooted in local entrepreneurs. Therefore, the behavior choice of entrepreneurs in the field of law and artificial intelligence was analyzed from the perspective of entrepreneurs’ psychological understanding. Entrepreneurs’ psychological understanding will produce changes in entrepreneurial behavior choices. Then, a model was built to mainly discuss the influencing factors of entrepreneurial behavior and its relationship mechanism. From the perspective of entrepreneurs’ psychological understanding and entrepreneurial opportunities, their effects on entrepreneurial behavior were explored, and the relationship between entrepreneurial opportunities and entrepreneurs’ psychological understanding was discussed. This investigation was conducted in the form of the questionnaire survey (Chen, 2018). The data were collected by sending emails, and statistical software was used to analyze the correlation between the factors in this investigation.

## Literature Review

Entrepreneurship is of great significance for the economic and social development of a country and region. The Chinese government and all sectors of society pay close attention to the development of entrepreneurial activities and strive to create a good environment to promote mass entrepreneurship and innovation. Furthermore, the vitality of hundreds of millions of market microsubjects is stimulated, and a new engine for economic development is created. In recent years, the market environment in China has been continuously improved, and entrepreneurial activities have been increasing, and the entrepreneurial economy has been comprehensively developed. Entrepreneurship has become a common practice among all people.

In response to the behavior of entrepreneurs, worldwide scholars have carried out different degrees of investigations. Miralles et al. (2017) explored the relationship between entrepreneurs’ personal beliefs and intentions as well as the relationship between entrepreneurial intentions and entrepreneurial behavior. The results show that participating in entrepreneurial activities can change the intentions of entrepreneurs. In addition, these influences depend on the age of the individual. It makes a positive contribution to exploring the reverse causal relationship between actual behavior and personal intention (Miralles et al., 2017). Sultana et al. (2019) explored the entrepreneurial behavior of IT freelancers and found relevant factors that would affect the entrepreneurial performance of IT freelancers (Sultana et al., 2019). Kim and Lee (2017) explored the success rate of entrepreneurial companies based on entrepreneurial behavior characteristics. The results show that entrepreneurial policy support has a positive effect on entrepreneurs’ behavioral characteristics. The behavioral characteristics of entrepreneurial opportunities and the use of entrepreneurial opportunities have certain effects on company performance (Kim and Lee, 2017). Guan et al. (2019) investigated the key factors influencing the innovative behavior of a new generation of entrepreneurs. The results show that the personal characteristics and educational background of new-generation entrepreneurs can affect their perception of innovation. Understanding innovation content and social capital will affect innovation behavior. Innovation behavior will further lead to changes in organizational performance (Guan et al., 2019). From the perspective of industrial development, with the development of the Internet, competition in the legal industry has become increasingly fierce, and the scale of law firms is gradually expanding. However, the personal field of legal services is developing in a small and refined direction. Therefore, China’s law firms are polarized and often lack cases. Since the field of artificial intelligence has only developed in recent years, there are few investigations on the direction of entrepreneurship. Therefore, certain field innovations are carried out based on previous works. The changing factors of psychological understanding and behavior choice of entrepreneurs are explored in the field of law and artificial intelligence (Zhou and Wu, 2018).

In summary, there are many researches in this field that discuss the psychological factors of entrepreneurs. Entrepreneurial understanding and entrepreneurial will directly affect innovation and entrepreneurial behavior. The innovation and entrepreneurial behavior will further cause changes in organizational performance. The form of this investigation is mainly based on questionnaires. Through questionnaire survey, the psychological understanding and behavior choice of entrepreneurs in related industries were analyzed and discussed. The innovation is that it is more targeted. The investigation was conducted for people in the law and artificial intelligence industries.

## Materials and Methods

### Entrepreneurial Behavior

At present, the definition of entrepreneurial behavior in academic circles is relatively vague. The main vagueness lies in the conceptual ambiguity between entrepreneurial activity and entrepreneurial behavior, as well as the description of entrepreneurial behavior is often carried out by the establishment of successful companies. The concept of entrepreneurial activities is relatively broad, which can be individuals and enterprises. The entrepreneurial behavior only targets individuals or entrepreneurial teams. In the investigation, the two are considered different. In addition, the entrepreneurial behavior of the entrepreneurial team is greatly different from corporate behavior. Corporate behavior is a description of the behavior of individuals in the enterprise, and entrepreneurial behavior is a description of the causes of entrepreneurial teams and entrepreneurs’ practices (Jiang et al., 2016; Martin and Omrani, 2019).

In this investigation, entrepreneurial behavior refers to the actions or decisions made by entrepreneurs or entrepreneurial teams related to the survival of the enterprise during the creation of a new enterprise. It does not include the operational behavior of existing enterprises (Shen et al., 2019).

In the process of creating new enterprises, entrepreneurial behavior will follow the entrepreneurial process to produce corresponding changes. Thus, entrepreneurial behavior is a dynamic process. In the process of entrepreneurship, changes in entrepreneurial behavior may occur every moment, which may affect the success of an enterprise’s entrepreneurship or even attribute differences (Wang et al., 2019).

### Entrepreneurial Opportunities

Entrepreneurial opportunities mainly refer to more attractive and more durable business opportunities that are conducive to entrepreneurship. It is attractive, durable, and timely. Entrepreneurial opportunities are situations where new products, new services, new raw materials, and new organizational methods can be introduced and sold at a price higher than the cost. Entrepreneurial opportunities are a new “Means–End” relationship, which can introduce new products, new services, new raw materials, new markets, or new organizational methods for economic activities (Qian et al., 2018). Based on this, entrepreneurs can provide customers with valuable products or services and benefit themselves (Zhu et al., 2019).

There are currently two factions. One side believes that entrepreneurial opportunities exist independently of entrepreneurs and need to be discovered by entrepreneurs from the outside environment. The other side holds that entrepreneurial opportunities are closely related to the entrepreneurs themselves, and their acquisition is based on the entrepreneurs’ perception and understanding of the outside world (Araslanov and Zelinskaya, 2018). In the investigation, the entrepreneurial opportunity ability is defined as the entrepreneur’s search and perception of various information in the market. Eventually, potential entrepreneurial opportunities are identified in the market, and the appropriate entrepreneurial method is chosen to start a business. The entrepreneurial opportunity abilities in the paper mainly include three abilities: opportunity search, opportunity recognition, and opportunity evaluation (Chen, 2019).

Opportunity search mainly reflects the sensitivity of entrepreneurs to outside information, that is, whether they can search for information that is beneficial to themselves from a large amount of information and collect the information. Opportunity recognition mainly reflects the ability of entrepreneurs to judge whether the collected information has a market value. Opportunity evaluation mainly reflects the judgment of entrepreneurs on the relationship between opportunities and themselves when facing opportunities, evaluating whether the opportunities are beneficial to their enterprises (Pallesen, 2018).

### Psychological Understanding of Entrepreneurs

The psychological understanding of entrepreneurs is inextricably linked to the previous experience, so the psychological understanding of entrepreneurs is described from three perspectives–educational experience, entrepreneurial experience, and industrial experience. Educational experience mainly refers to the previous education of entrepreneurs, and it mainly reflects the theoretical psychology of entrepreneurs. Entrepreneurial experience and industrial experience mainly describe entrepreneurs’ entrepreneurial capital psychology (Shen et al., in press).

### Model Building

**FIGURE 1:**
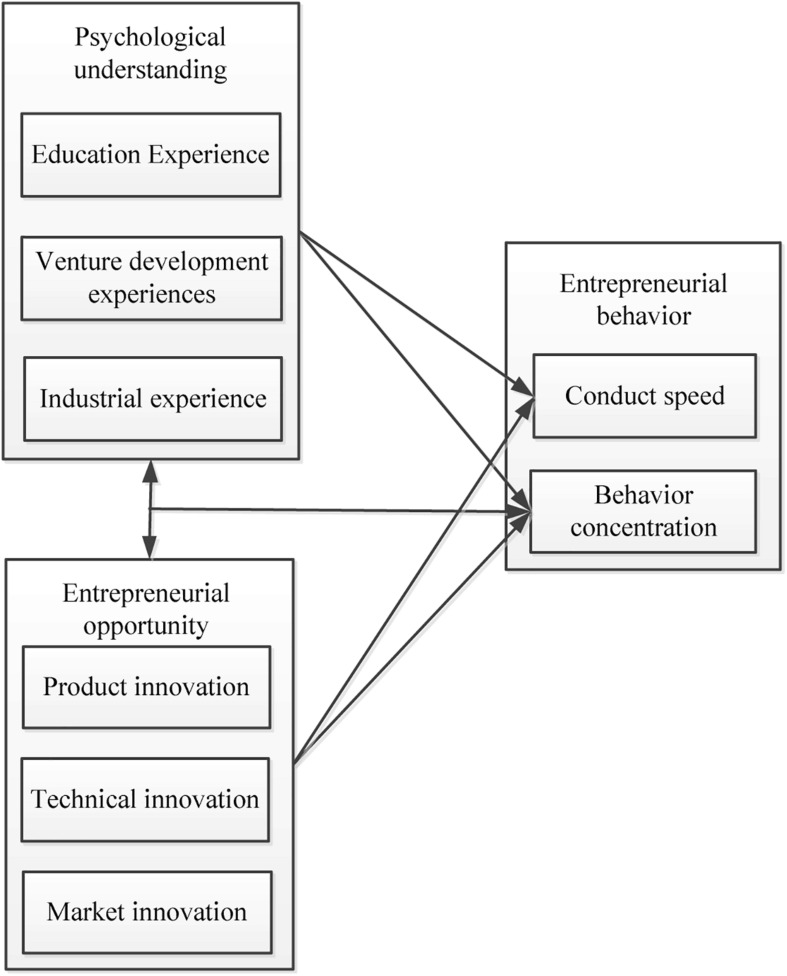
Research design model.

Based on the entrepreneurs’ psychological understanding producing the changes in the choice of entrepreneurial behavior, a model was built to mainly discuss the influencing factors of entrepreneurial behavior and its relationship mechanism. From two aspects of entrepreneurs’ psychological understanding and entrepreneurial opportunities, the impact on entrepreneurial behavior as well as the relationship between entrepreneurial opportunities and entrepreneurs’ psychological understanding were explored (Obschonka et al., 2018; Shen et al., 2019). The designed model is shown in Figure 1.

There is a certain relationship between entrepreneurs’ behavior choice and psychological understanding with entrepreneurial opportunities. In addition, the psychological understanding of entrepreneurs is closely related to their previous experiences. The entrepreneurial opportunities of entrepreneurs will affect their psychological understanding and experience. As can be seen from Figure 1, the relationship between entrepreneurs’ psychological understanding and entrepreneurial opportunities was first explored. After that, the investigations on the effects of entrepreneurs’ psychological understanding and entrepreneurial opportunities on entrepreneurial behavior were conducted.

### Research Object Screening

The research object consists of entrepreneurs in the field of law and artificial intelligence. The relevant industry practitioners and entrepreneurs were found from the major job search sites, and questionnaires were distributed to them by email. A total of 300 questionnaires were distributed, including 150 in the legal industry and 150 in the artificial intelligence industry. A total of 134 questionnaires were recovered in the legal industry, and 125 questionnaires were recovered in the artificial intelligence industry. The questionnaire recovery rates were 89.3 and 83.3%, respectively. The overall questionnaire recovery rate was 86.3%.

### Questionnaire Design

The questionnaire is mainly divided into two parts. Personal information about the entrepreneur was collected for the first part, and entrepreneurial information was collected for the second part. A total of two questionnaires were designed: one for the entrepreneurs in the legal industry (hereinafter referred to as “Questionnaire 1”) and the other for the entrepreneurs in the artificial intelligence industry (hereinafter referred to as “Questionnaire 2”). The questionnaire was sent online to five cities, including Beijing and Shanghai.

Questionnaires 1 and 2 were designed using the Likert 5 score method, including the dimensions of entrepreneurs’ psychological understanding and entrepreneurial opportunities mentioned above. Respondents made choices from “completely compliant” to “completely non-compliant” according to their situation, scoring 1–5 points, respectively (Ye and Zhu, 2019). Questionnaires 1 and 2 were almost the same in the content of the survey, but they were oriented different industries. Therefore, the two questionnaires were only slightly different in the collection of personal information of entrepreneurs.

### Questionnaire Reliability Analysis

The reliability analysis of the questionnaire is also the responsibility analysis of the questionnaire. Responsibility is to verify the stability and consistency of the questionnaire test results. In terms of verification results, the higher the reliability of the questionnaire, the smaller the test error of the questionnaire (Zhou et al., 2018). Reliability indicators are mostly expressed in correlation coefficients, which can be roughly divided into three categories: stability coefficients (consistency across time), equivalence coefficients (consistency across forms), and intrinsic consistency coefficients (consistency across projects). There are four main methods for reliability analysis: retest reliability, replica reliability, halved reliability, and α reliability coefficient.

The Cronbach α reliability coefficient is most commonly used. The equation is shown below.

(1) $$\alpha ={\left(k/\left(k-1\right)\right)}^{*}\,\left(1-\left(\sum S\,i\,\wedge \,2\right)/S\,T\,\wedge \,2\right)$$

where *k* is the total number of items in the scale, *Si*^2 the in-question variance of the *i*th score, and *ST*^2 the variance of the total score of all items. It can be seen from the equation that the α coefficient evaluates the consistency between the scores of each item in the scale, which belongs to the intrinsic consistency coefficient. This method is suitable for the reliability analysis of attitude and opinion questionnaires (scales).

The reliability coefficient of the total scale is preferably above 0.8, which is acceptable between 0.7 and 0.8.

### Questionnaire Validity Analysis

Validity analysis refers to the analysis of scales to achieve the accuracy of measurement indicators. There are many methods of validity analysis, often using item analysis, independent criterion measurement validity analysis, and factor analysis (Netto et al., 2020). Item analysis is mainly to measure the difficulty and discrimination of various items in the scale, to select a moderate scale with higher discrimination as the effective scale. The independent criterion measurement analysis method mainly uses a certain independent validity as the criterion and basis for validity analysis.

The KMO test statistic is an index used to compare simple correlation coefficients and partial correlation coefficients between variables. It is mainly used for factor analysis of multivariate statistics (Cui, 2019). The KMO metrics: above 0.9 means very suitable, 0.8 means suitable, 0.7 means general, 0.6 means not very suitable, and below 0.5 means extremely unsuitable. When the sum of squares of simple correlation coefficients among all variables is much larger than the sum of squares of partial correlation coefficients, the KMO value is close to 1. The closer the KMO value is to 1, the stronger the correlation between the variables, and the more suitable the original variables are for factor analysis. When the sum of squares of simple correlation coefficients between all variables is close to 0, the KMO value is close to 0. The closer the KMO value is to 0, the weaker the correlation is between the variables, and the less suitable the original variables are for factor analysis.

### Statistical Methods

After a quality inspection of the original data obtained from the survey was conducted, Microsoft Excel was used for data entry. Data analysis was performed using IBM SPSS 26.0 and AMOS 24.0 software. General data were expressed as mean and standard deviation, composition ratio, and frequency. Independent-samples *t* test was used for the comparison of measurement data of two groups. Chi-square test (Crosstabs) was used to compare the count data between the two groups. If *P* < 0.05, the difference was considered statistically significant.

## Results

### Descriptive Analysis of the Sample

**FIGURE 2:**
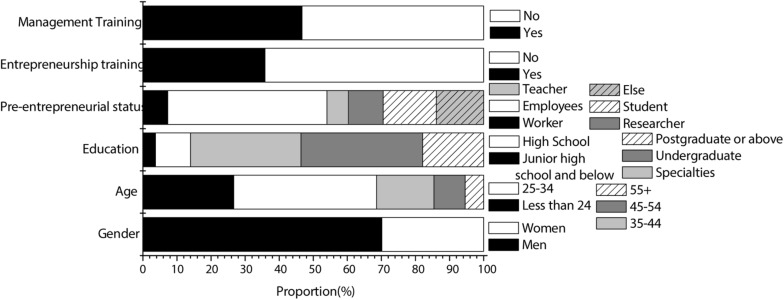
Sample of artificial intelligence industry.

**FIGURE 3:**
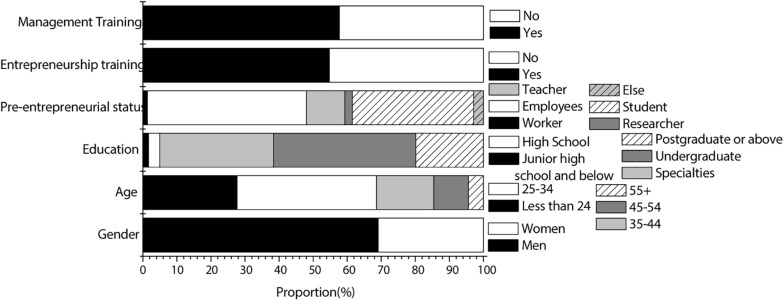
Sample of the law industry.

According to the questionnaire, artificial-intelligence-related industry personnel surveyed were mainly male, mostly the generation from the 1980s and 1990s. The education level of interviewees was mainly junior college and undergraduate. Most of the interviewees were employees of enterprises before starting the business. Most people started a business without receiving entrepreneurship training. In addition, those who had received management training were almost the same as those who had not. The specific results of the survey of entrepreneurs are shown in Figure 2. The law-related industry personnel surveyed by the investigation were also mainly male, mostly the generation after the 1980s and 1990s. The education level was mainly undergraduate and graduate. Most of the interviewees were employees of enterprises before starting the business, but a large part of them started their business after graduation. The majority of people had received entrepreneurship training. In addition, those who had received management training were almost the same as those who had not. The specific results of the survey of entrepreneurs are shown in Figure 3.

As can be seen from Figures 2, 3, the majority of entrepreneurs in the legal and artificial intelligence industries are male. Different from the artificial intelligence industry, among the entrepreneurs in the law industry, the number of former teachers has increased significantly. It may be because the teachers of the law profession are more likely to start relevant businesses abroad. The entrepreneurial number of students in the law industry is also relatively high, which may be due to the large employment pressure of the law industry.

### Analysis of Questionnaire Reliability and Validity

Through the investigation, the Cronbach α reliability coefficient of the product innovation factor in the questionnaire is 0.769. Cronbach α reliability coefficient of the technological innovation factor is 0.792. Cronbach α reliability coefficient of the market innovation factor is 0.696. Cronbach α reliability coefficient of the educational experience factor is 0.784. Cronbach α reliability coefficient of the entrepreneurial experience factor is 0.911. Cronbach α reliability coefficient of the industrial experience factor is 0.901. The item-overall correlation coefficients in the scale are >0.4, which proves that the scale has good reliability.

After validity analysis, the results show that the value of KMO in this questionnaire is 0.819, with a significant level of 0.000. The value of KMO in Questionnaire 1 is 0.797, with a significant level of 0.000. It indicates that the relationship between the variables in this questionnaire is good, which is suitable for factor analysis.

The principal component analysis shows that there are six initial eigenvalues > 1, and the accumulation of initial eigenvalues reaches 69.883%. The factor has more information, which is higher than the general standard of validity analysis.

### Correlation Analysis of Entrepreneurs’ Psychological Understanding and Behavior Choice

Through the analysis of Questionnaires 1 and 2, the correlation coefficient matrix of entrepreneurs’ psychological understanding and behavior choice is obtained, as shown in Table 1.

**TABLE 1:** The correlation coefficient matrix of entrepreneurs’ psychological understanding and behavior choice.

Variable	1	2	3	4	5	6	7
Gender	1	0.071	–0.049	0.091	0.057	–0.063	–0.092
Age	0.069	1	0.0135**	0.0162**	0.324**	−0.312**	−0.335**
Educational experience	–0.051	−0.179**	1	−0.225*	0.096*	0.382*	0.201
Entrepreneurial experience	0.089	0.181**	−0.102*	1	0.322	0.341*	0.061
Industrial experience	0.060	–0.034	0.101*	–0.059	1	0.441	−0.079*
Behavior speed	0.076	−0.249**	0.033	–0.015	–0.015	1	0.302**
Behavior concentration	–0.083	−0.301**	–0.011	0.056	–0.086	0.601**	1

Above the diagonal is the report of law industry personnel, and below the diagonal is the report of artificial intelligence industry personnel. *p < 0.05, **p < 0.01.

According to Table 1, the behavior speed and concentration of entrepreneurs in the legal industry are negatively correlated with the age of entrepreneurs, and the difference is statistically significant. There is a positive correlation between the educational experience and the entrepreneurs’ behavior speed, as well as the difference is statistically significant. There is a positive correlation between entrepreneurial experience and entrepreneurs’ behavior speed, as well as the difference is statistically significant. The behavior concentration of entrepreneurs is negatively correlated with the entrepreneurs’ industrial experience, and the difference is statistically significant.

The behavior speed and concentration of entrepreneurs in the artificial intelligence industry are negatively correlated with the age of entrepreneurs, and the difference is statistically significant. There is a positive correlation between the behavior concentration and speed of entrepreneurs, as well as the difference is statistically significant.

### Correlation Analysis of Entrepreneurs’ Psychological Understanding and Innovation Opportunities

Through the analysis of Questionnaires 1 and 2, the correlation coefficient matrix of entrepreneurs’ psychological understanding and innovation opportunities is obtained, as shown in Table 2.

**TABLE 2:** The correlation coefficient matrix of entrepreneurs’ psychological understanding and innovation opportunities.

Variable	1	2	3	4	5	6	7	8
Gender	1	0.071	–0.049	0.091	0.057	0.031*	0.059	0.041
Age	0.069	1	0.0135**	0.0162**	0.324**	0.024*	0.235**	−0.033*
Educational experience	–0.051	−0.179**	1	0.225*	0.096*	0.310	0.032*	0.032**
Entrepreneurial experience	0.089	0.181**	−0.102*	1	0.322	0.112	0.103	0.021
Industrial experience	0.060	–0.034	0.101*	–0.059	1	0.031	0.310*	0.031**
Product innovation	0.020	0.201**	0.101	0.072	0.031	1	0.311**	0.024*
Technological innovation	0.061	0.211**	–0.051	0.071	0.136**	0.152**	1	0.323*
Market innovation	0.052	–0.032	0.031	0.013	0.021	0.312**	0.173**	1

Above the diagonal is the report of law industry personnel, and below the diagonal is the report of artificial intelligence industry personnel. *p < 0.05, **p < 0.01.

According to Table 2, the educational experience, entrepreneurial experience, and industrial experience of entrepreneurs in the legal industry are positively correlated with the age of entrepreneurs, and the difference is statistically significant. It is almost in line with common sense that entrepreneurs will have more experience in all aspects as they grow older. The educational experience is negatively correlated with the entrepreneurial experience and industrial experience of entrepreneurs, as well as the difference is statistically significant. There is a certain difference between men and women in the field of product innovation, and the difference is statistically significant. The educational experience of entrepreneurs is positively correlated with technological and market innovations, as well as the difference is statistically significant. The age of entrepreneurs is positively correlated with technological innovation but negatively correlated with market innovation.

The age of entrepreneurs in the artificial intelligence industry is negatively correlated with educational experience and positively correlated with entrepreneurial experience. The educational experience of entrepreneurs is negatively correlated with entrepreneurial experience and positively correlated with industrial experience. The entrepreneurs’ industrial experience is positively correlated with technological innovation.

### Correlation Analysis of Entrepreneurs’ Behavior Choice and Innovation Opportunities

Through the analysis of Questionnaires 1 and 2, the correlation coefficient matrix of entrepreneurs’ behavior choice and innovation opportunities is obtained, as shown in Table 3.

**TABLE 3:** The correlation coefficient matrix of entrepreneurs’ behavior choice and innovation opportunities.

Variable	1	2	3	4	5	6	7
Gender	1	0.071	–0.063	0.092	0.031*	0.059	0.041
Age	0.069	1	−0.312**	−0.335**	0.024*	0.235**	−0.033*
Behavior speed	0.076	−0.249**	1	0.302**	0.319*	0.154**	0.211*
Behavior concentration	–0.083	−0.301**	0.601**	1	0.411**	0.068*	0.213**
Product innovation	0.020	0.201**	0.321**	0.413*	1	0.311**	0.024*
Technological innovation	0.061	0.211**	0.445**	0.442*	0.152**	1	0.323*
Market innovation	0.052	–0.032	0.511**	0.305**	0.312**	0.173**	1

Above the diagonal is the report of law industry personnel, and below the diagonal is the report of artificial intelligence industry personnel. *p < 0.05, **p < 0.01.

According to Table 3, the behavior speed of entrepreneurs in the legal industry is positively correlated with product innovation, technological innovation, and market innovation. In addition, the behavior concentration of entrepreneurs is positively correlated with product innovation, technological innovation, and market innovation.

The behavior speed of entrepreneurs in the artificial intelligence industry is positively correlated with product innovation, technological innovation, and market innovation. In addition, the behavior concentration of entrepreneurs is positively correlated with product innovation, technological innovation, and market innovation.

## Discussion

At present, China supports innovation and entrepreneurship in various industries. In addition, the legal and artificial intelligence industries are strongly supported by China. Therefore, the targeted investigation was conducted on the above two directions (Zheng et al., 2020).

Through the investigation, it can be seen that the entrepreneurs’ psychological understanding and entrepreneurial experience in the law industry show positive correlation characteristics and also have a positive correlation with the entrepreneurs’ behavior speed. It indicates that the entrepreneurs’ psychological understanding will directly affect their behavior speed. The entrepreneurial experience of entrepreneurs is also positively correlated with their behavior speed, which indicates that the psychological understanding of entrepreneurs will also impact their behavior speed by affecting entrepreneurial opportunities (Wu et al., 2020). The concentration of entrepreneurs’ behavior will be affected by their entrepreneurial experience, which shows a negative correlation. The behavior speed and concentration of entrepreneurs in the artificial intelligence industry are negatively correlated with the age of entrepreneurs, and the difference is statistically significant. However, the entrepreneurial behavior and psychological understanding do not show correlation. The behavior speed and concentration of entrepreneurs in the artificial intelligence industry are positively correlated with product innovation, technological innovation, and market innovation.

Based on that entrepreneurs’ psychological understanding will produce changes in entrepreneurial behavior choices, a model is built to mainly discuss the influencing factors of entrepreneurial behavior and its relationship mechanism. From the perspective of entrepreneurs’ psychological understanding and entrepreneurial opportunities, their effects on entrepreneurial behavior are explored. In addition, the relationship between entrepreneurial opportunities and entrepreneurs’ psychological understanding is discussed. The results show that the behavior choice of entrepreneurs in the legal field is related to psychological understanding and innovation opportunities, as well as innovation opportunities are related to psychological understanding. It indicates that innovation awareness can not only directly affect entrepreneurs’ behavior choices but also indirectly affect entrepreneurs’ behavior choices by affecting entrepreneurs’ psychological understanding (Wu and Wu, 2019).

The behavior choice of entrepreneurs in the field of artificial intelligence does not show a trend directly affected by psychological understanding, but it is related to innovation ability. It can be seen from the above results that the behavior choice of entrepreneurs in the legal industry is related to their psychological understanding. It means that when entrepreneurs make certain behavior decisions, they should conduct certain discussions with others. Each individual’s psychological understanding is different. Therefore, each individual’s views on behavior also have certain differences. The corresponding discussion can better avoid the implementation of wrong decisions.

## Conclusion

Related practitioners in the legal and artificial intelligence industries were explored to obtain the relationship between entrepreneurs’ psychological understanding and behavior choice. The results show that the behavior choice of entrepreneurs in the legal industry is highly correlated with psychological understanding. The entrepreneurs’ psychological understanding and behavior choice in the field of artificial intelligence show an indirect correlation through indirect factor innovation opportunities. Although some results have been obtained, there are still some shortcomings. Due to the time limitation, the sample scale is small, and a follow-up research cannot be carried out. What is more, the questionnaire survey is subjective and lacks some objective data for illustration. Therefore, a follow-up research is needed to verify the results.

## Data Availability Statement

The raw data supporting the conclusions of this article will be made available by the authors, without undue reservation.

## Ethics Statement

The studies involving human participants were reviewed and approved by Southeast University Ethics Committee. The patients/participants provided their written informed consent to participate in this study.

## Author Contributions

The author confirms being the sole contributor of this work and has approved it for publication.

## Conflict of Interest

The author declares that the research was conducted in the absence of any commercial or financial relationships that could be construed as a potential conflict of interest.
